# Applying Radiomics to Predict Outcomes in Patients with High-Grade Retroperitoneal Sarcoma Treated with Preoperative Radiotherapy

**DOI:** 10.3390/jimaging11120450

**Published:** 2025-12-15

**Authors:** Adel Shahnam, Nicholas Hardcastle, David E. Gyorki, Katrina M. Ingley, Krystel Tran, Catherine Mitchell, Sarat Chander, Julie Chu, Michael Henderson, Alan Herschtal, Mathias Bressel, Jeremy Lewin

**Affiliations:** 1 Department of Medical Oncology, Peter MacCallum Cancer Center, Melbourne 3000, Australia; 2 Department of Radiation Oncology, Peter MacCallum Cancer Center, Melbourne 3000, Australiakrystel.tran@petermac.org (K.T.);; 3 Department of Surgical Oncology, Peter MacCallum Cancer Center, Melbourne 3000, Australia; 4 Sir Peter MacCallum Department of Oncology, University of Melbourne, Melbourne 3000, Australia; 5 Department of Anatomical Pathology, Peter MacCallum Cancer Center, Melbourne 3000, Australia; 6 Centre for Biostatistics and Clinical Trials, Peter MacCallum Cancer Center, Melbourne 3000, Australia; 7 Victorian Adolescent and Young Adult Cancer Service, Melbourne 3000, Australia

**Keywords:** retroperitoneal sarcomas, radiomics, predictive model

## Abstract

Retroperitoneal sarcomas (RPS) are rare tumours, primarily treated with surgical resection. However, recurrences are frequent. Combining clinical factors with CT-derived radiomic features could enhance treatment stratification and personalization. This study aims to assess whether radiomic features provide additional prognostic value beyond clinicopathological features in patients with high-risk RPS treated with preoperative radiotherapy. This retrospective study included patients aged 18 or older with non-recurrent and non-metastatic RPS treated with preoperative radiotherapy between 2008 and 2016. Hazard ratios (HR) were calculated using Cox proportional hazards regression to assess the impact of clinical and radiomic features on time to event outcomes. Predictive accuracy was assessed with c-statistics. Radiomic analysis was performed on the high-risk group (undifferentiated pleomorphic sarcoma, well-differentiated/de-differentiated liposarcoma or grade 2/3 leiomyosarcoma). Seventy-two patients were included, with a median follow-up of 3.7 years, the 5-year overall survival (OS) was 67%. Multivariable analysis showed older age (HR: 1.3 per 5-year increase, *p* = 0.04), grade 3 (HR: 180.3, *p* = 0.02), and larger tumours (HR: 4.0 per 10 cm increase, *p* = 0.02) predicted worse OS. In the higher-risk group, the c-statistic for the clinical model was 0.59 (time to distant metastasis (TDM)) and 0.56 (OS). Among 27 radiomic features, kurtosis improved OS prediction (c-statistic 0.69, *p* = 0.013), and Neighbourhood Gray-Tone Difference Matrix (NGTDM) busyness improved it to 0.73 (*p* = 0.036). Kurtosis also improved TDM prediction (c-statistic 0.72, *p* = 0.023). Radiomic features may complement clinicopathological factors in predicting overall survival and time to distant metastasis in high-risk retroperitoneal sarcoma. These exploratory findings warrant validation in larger, multi-institutional studies.

## 1. Introduction

Retroperitoneal sarcomas (RPS) are a group of rare tumours that represent approximately 0.2% of all malignancies and 10–15% of all soft-tissue sarcomas (STS) [1,2]. Approximately half of RPS are intermediate or high-grade lesions with the most common histologic subtypes being de-differentiated liposarcoma, leiomyosarcoma, and undifferentiated pleomorphic sarcoma (UPS). For patients with localized disease, surgical resection is the mainstay of treatment with the aim of microscopic negative margins [3]. Unfortunately, recurrences are common and outcomes remain poor with a 5-year OS of approximately 60% [3]. When compared to other STS, RPS have a worse overall outcome compared with matched histologies in the extremity potentially related to the difficulties with its anatomical location and large size at presentation [4]. Improving the outcomes for RPS has remained a significant challenge with a major study reporting no significant benefits from neoadjuvant radiotherapy [5]. However, RPS are heterogeneous leading to variability in potential response to treatments, and, therefore, identifying subgroups who might benefit from radiotherapy or other treatment strategies remains crucial. Furthermore, predicting distant recurrences is crucial, as these patients may benefit more from neo/adjuvant systemic therapy to address micrometastatic disease. Improved methods for predicting recurrence and survival are thus required for optimizing treatment stratification and personalizing care.

While several clinical prognostic factors have been established (e.g., resectability, age, tumour size, grade, high-risk histology (UPS, well-differentiated/de-differentiated liposarcoma or grade 2/3 leiomyosarcoma), multifocality, histologic organ invasion, surgery in a high volume centre), the predictive power of current nomograms remains limited, with reported C-indices ranging from 0.71 to 0.74 [2,6,7]. Since computed tomography (CT) is a cornerstone of RPS diagnosis, the development of models that integrate existing clinical factors with CT-derived radiomic features holds promise for improving prediction accuracy and supporting treatment decision-making [8].

Radiomics involves advanced analysis of quantitative features extracted from conventional imaging, offering potential for diagnostic, prognostic, or predictive applications across diverse tumour types like lung cancer, head and neck cancer, glioblastoma, rectal cancer, hepatocellular carcinoma, and germ cell tumour [9,10,11,12,13]. These non-invasive imaging tools hold great potential to augment clinical prediction algorithms for enhanced patient prognostication and treatment selection. Radiomics has demonstrated evolving clinical utility across multiple oncologic settings, including tumour staging, early diagnosis, disease differentiation, prognosis prediction, and treatment response assessment [14].

The main radiomic features used for analysis include first-order, the Gray level co-occurrence matrix (GLCM), the Gray Level Dependence Matrix (GLDM), the Gray Level Run Length Matrix (GLRLM), the Gray Level Size Zone Matrix (GLSZM), the Neighbourhood Gray-Tone Difference Matrix (NGTDM), and shape features [15]. First-order features, such as mean, median, standard deviation, and kurtosis describe the distribution of pixel intensities within a region of interest. The GLCM quantifies texture by assessing the spatial relationship between pixel intensities, providing metrics like contrast and homogeneity. The GLDM evaluates the dependence of a pixel on its neighbours, capturing granularity in texture. GLRLM measures the length of consecutive pixels with the same intensity, highlighting texture patterns. The GLSZM assesses the size of homogeneous zones, reflecting structural information. The NGTDM evaluates the difference between a pixel and its neighbours, indicating local texture variations. Shape features describe the geometric properties of a tumour, such as volume and surface area [15]. These radiomic features are crucial in radiomic analysis as they provide comprehensive information on tumour heterogeneity, aiding in diagnosis, prognosis, and treatment response evaluation.

Several studies have explored the use of radiomics in soft-tissue sarcomas [16,17,18]. Notably, the RADSARC-R study reported a validated radiomics model with high accuracy in predicting RPS histology and grade [19]. However, research on the predictive performance of combined radiomic and clinical models for relapse and survival in RPS remains scarce. This study aims to assess whether radiomic features provide additional prognostic value beyond clinicopathological features in patients with high-risk RPS treated with preoperative radiotherapy. This, in turn, could potentially identify high-risk patients who might benefit from the addition of chemotherapy.

## 2. Methods

This is a single-institutional retrospective study of patients with RPS who were treated with preoperative radiotherapy at the Peter MacCallum Cancer Centre between 1 January 2008 and 31 December 2016. After receiving institutional ethics approval (PMCC 18/168R), patients were identified via institutional health records. Due to the retrospective nature of the study, informed consent was waived by institutional ethics review. The study population included patients aged 18 years or above and availability of radiation therapy treatment planning computed tomography (CT) imaging. Patients had to have received neoadjuvant radiotherapy (conformal radiotherapy was given to a dose of 50.4 Gy in 1.8 fractions, daily for 5 days over 6 weeks) and had complete, curative-intent tumour resection performed at the Peter MacCallum Cancer Centre. This radiation dose was the institutional standard at the time of data collection. Patients who had history of any other malignancies diagnosed in the last 5 years or had a recurrent RPS were excluded.

Pre-treatment clinical and pathological data were obtained from the health records. Radiomics features were developed from radiation therapy treatment planning CT images. High-risk disease was defined as UPS, differentiated liposarcoma, or grade 2/3 leiomyosarcoma. Radiomics analysis was performed on images acquired for patients in this high-risk group.

### 2.1. Segmentation

The gross tumour volume (GTV) as used for RT treatment planning was used as the starting point for all segmentations. The GTV was reviewed by a member of the study team (KT) and refined to exclude non-tumour anatomy such as bone, bowel, and other organs. Where there was ambiguity in the tumour boundary, a senior radiation oncologist (SC) reviewed the segmentation and adjusted as necessary. Images were reviewed at the location of the GTV to determine any artefacts which may impact radiomics feature extraction.

### 2.2. Radiomics Feature Extraction

CT imaging and segmentations were exported in DICOM format. Images were converted to meta image format, and the GTV segmentation was converted to a binary mask using Plastimatch (v1.8; open-source software; Massachusetts General Hospital, Boston, MA, USA). PyRadiomics (v2.1.2; open-source software; Harvard Medical School, Boston, MA, USA) was used to extract 105 features from the original image; first order statistics, shape, gray level co-occurrence matrix, gray level run length matrix, gray level size zone matrix, neighbouring gray tone difference matrix, and gray level dependence matrix features were extracted. An extraction intensity bin width was set at 10 Hounsfield units, images were resampled to 1 × 1 × 1 mm, and no image intensity normalization was performed [20,21]. Intensity normalization was not applied because the CT images were acquired using a uniform tube potential and largely identical scanner and reconstruction parameters, resulting in a homogeneous dataset where additional normalization was unlikely to alter feature behaviour. Feature extraction was performed on a Dell Precision 5680 workstation.

Since the number of radiomics features was large (105, Supplementary Table S1), a dimensionality reduction step was performed as follows. All pairs of features with an absolute value of Spearman’s rank correlation > 0.8 were identified. Then, iteratively, the following steps were followed to determine inclusion in the final feature set to be used for modelling. All features were marked as ‘yet to be classified’ for inclusion. The feature yet to be classified with the smallest mean absolute correlation with all other features was marked for inclusion in the final feature set. All other features having absolute value of Spearman’s rank correlation > 0.8 with that feature included in the previous step were marked for exclusion from the final feature set. This was repeated iteratively until all features were either classified for inclusion or exclusion. This procedure reduced the dimensionality of the radiomics feature set from 105 features to 27 features. This conservative, correlation-based approach was chosen to minimize multicollinearity and reduce the risk of model overfitting given the modest sample size and limited number of outcome events. Penalized regression techniques such as LASSO were considered but deemed unsuitable, as they typically require substantially larger datasets to produce stable and generalizable feature selection.

### 2.3. Statistical Analysis

Patient demographics, baseline characteristics, and treatment details were described using descriptive statistics. The Kaplan–Meier method was used to describe the time-to-event curves. Overall survival (OS) was defined by time from surgery until death from any cause. Relapse free survival (RFS) was defined as time from surgery until disease relapse (local or distant) or death. Time to local recurrence (TLR) and time to distant metastasis (TDM) were defined as time from surgery until local relapse or distant disease progression, respectively. Cox proportional hazard models were used to assess the impact of clinical and radiomic features on time-to-event endpoints. Multivariable analysis of radiomic features was carried out for each radiomic feature adjusting for age at time of surgery and maximum tumour size. Hazard ratios, 95% confidence intervals, likelihood ratio *p*-values, and c-statistics were provided for each model. The assumption of proportional hazards was verified for each of the models. The presence of influential observations was checked for each of the models. Linearity for radiomic features was assessed. No adjustment for multiplicity was performed as the analysis was exploratory in nature. All statistical analyses were performed in R (v4.1.1; R Foundation for Statistical Computing, Vienna, Austria).

## 3. Results

**Figure 1:**
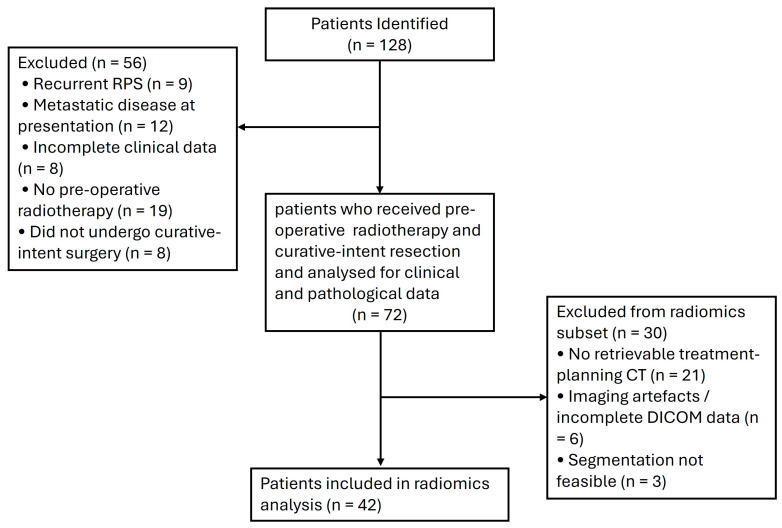
Study flow diagram.

Seventy-two patients met the inclusion criteria for this study, as outlined in the study flow diagram (Figure 1). Table 1 summarizes the demographic and clinical characteristics of the cohort. The median follow-up was 3.7 years, and the median age at diagnosis was 57 years (range, 31–86 years). Most patients were male (61%) and had an ECOG performance status of 0 (58%). WD-DDLPS was the most common histologic subtype (42%), and the median maximum tumour dimension on imaging was 132 mm (range, 23–360 mm). The majority of tumours were grade 2 or 3. Supplementary Table S2 provides a detailed distribution of tumour grades by histologic subtype. 

### 3.1. Recurrence and Survival Outcomes

**Figure 2:**
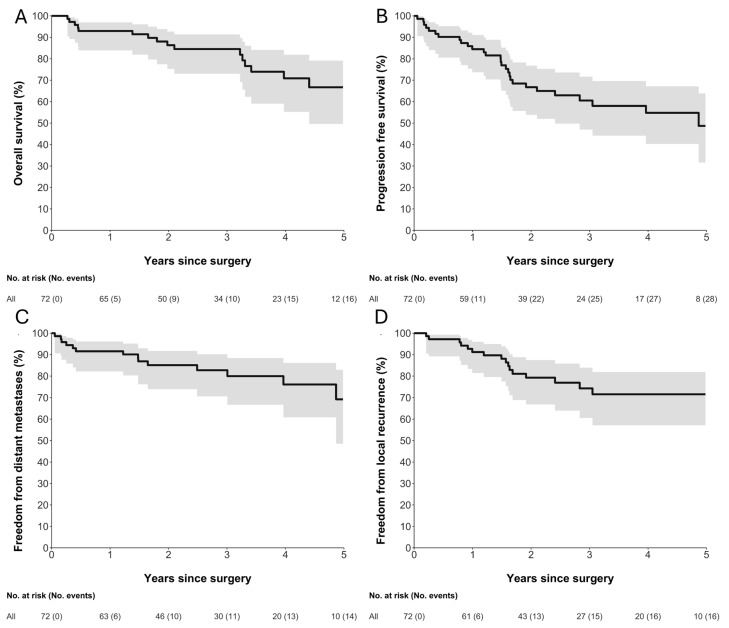
Kaplan–Meier estimates of (**A**) overall survival, (**B**) relapse-free survival, (**C**) freedom from distant metastases, and (**D**) freedom from local recurrence following surgery. Solid black lines represent Kaplan–Meier survival estimates, and grey shaded areas indicate 95% confidence intervals. Numbers at risk and cumulative numbers of events are shown below each plot. Time is measured in years since surgery.

Figure 2 illustrates the OS, RFS, TLR, and TDM for the study cohort. The 5-year landmark estimates were 67% for OS, 49% for RFS, 72% for TLR, and 69% for TDM. Median RFS and OS were 4.9 years (95% CI 2.4—not reached) and 7.4 years (95% CI 4.4—not reached), respectively. These estimates are based on a small number of patients at risk and should therefore be interpreted with caution due to limited precision.

### 3.2. Clinical Predictors of Survival

Table 2 summarizes the univariable and multivariable analyses of clinical variables associated with survival outcomes. Older age, higher grade, and larger tumour size were associated with worse overall survival. For RFS, older age remained the only variable significantly associated with poorer outcomes (Supplementary Table S3).

### 3.3. Radiomics

**Figure 3:**
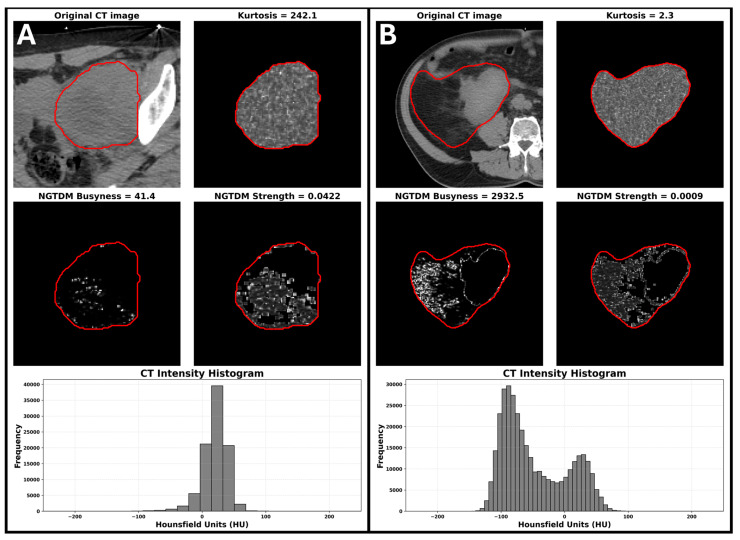
The visual interpretation of variations in radiomic features such as kurtosis and NGTDM. (**A**) Example of a patient with high kurtosis, low NGTDM busyness, and high NGTDM strength. (**B**) Example of a patient with low kurtosis, high NGTDM busyness, and low NGTDM strength. Red contours delineate the gross tumor volume used for radiomic feature extraction. The feature scores for the patient on the left were associated with improved OS while the feature scores for the patient on the right were associated with poorer survival.

Radiomics analysis was performed on 42 high-risk disease patients for whom imaging was available. Images were acquired on a Philips Brilliance Big Bore (n = 38), or a GE Discovery PET/CT (n = 4), all at 140 kV with 3 mm slice thickness. Given the very limited number of events (15 deaths and 13 distant failures in this subset), only age and maximum dimension were added to the Cox models with radiomic features. The c-statistic for the clinical model (incorporating age and maximum tumour dimension) for OS and TDM was 0.56 and 0.59. Table 3 summarizes the overall survival results for each radiomic features adjusted by tumour size and age (i.e., each line in the table corresponds to a different Cox model). Bold rows indicate radiomic features with an increase in c-statistic > 0.1 compared with the clinical model including age and tumor size alone . For TDM, kurtosis was the only radiomic feature that was predictive (c-statistic 0.72) (Supplementary Table S4). The visual interpretation of variations in radiomic features such as kurtosis and NGTDM are illustrated in Figure 3. In the context of liposarcomas, Figure 3A demonstrates a mass with very uniform density and low fat content resulting in high kurtosis, low NGTDM busyness, and high NGTDM strength, features associated with improved OS. Conversely, Figure 3B shows a mass with a high fat content, as well as a higher density component with low kurtosis, high NGTDM busyness, and low NGTDM strength, associated with poorer OS.

## 4. Discussion

This exploratory study evaluated the prognostic potential of radiomic features in addition to clinicopathological features in patients with high-risk RPS treated with preoperative radiotherapy. Age, maximum tumour dimension, and grade were associated with overall survival in the model with clinical variables alone. When incorporating radiomics features in patients with high-risk histology, some radiomic features such as NGTDM busyness and strength showed potential additional prognostic value for OS, worthwhile of further investigation in a larger study.

Our study aligns with prior research that identified grade, age, and tumour size as significant predictors of OS in RPS patients [2,22,23]. While our initial clinical model demonstrated a low c-statistic for predicting both OS (c-statistic 0.56), incorporating radiomic features improved its performance (c-statistics 0.72). Age, grade, and tumour size are well-established prognostic factors for RPS and are integrated into clinical prognostic tools like the Sarculator. This calculator quantifies the risk of recurrence and guides treatment decisions [23,24]. The Sarculator’s C-index for OS and RFS typically ranges from 0.60 to 0.70, indicating moderate predictability [25]. To our knowledge, no existing studies have explored the integration of radiomic features with the Sarculator or other clinicopathological models for RPS. This aspect represents a novel contribution of our investigation.

Prognostic tools such as Sarculator have improved outcome prediction in retroperitoneal and soft-tissue sarcomas but retain only moderate discriminative performance and would benefit from further refinement to support individualized prognostication [26]. Recent studies have proposed incorporating novel biomarkers, including radiomic, genomic, and molecular features to enhance these models. However, emerging evidence indicates that adding candidate biomarkers to Sarculator does not consistently improve prognostic accuracy, underscoring the need to evaluate modalities such as radiomics for genuine incremental value in risk prediction and clinical decision-making [27]. Kurtosis measures the intensity distribution of an image. High kurtosis indicates a distribution with a sharp peak and heavy tails, while low kurtosis suggests a flatter distribution with fewer extreme values. Tumours with higher kurtosis are more heterogeneous and potentially more aggressive [15,28]. The NGTDM filter applied to an image results in a matrix of the differences between the intensity of a voxel and the average of the surrounding voxels within a specified distance. NGTDM busyness measures the rate of intensity change within the image, with higher busyness reflecting rapid changes in intensity values [15,28]. NGTDM strength captures the consistency of gray levels throughout the CT image, where higher NGTDM strength indicates a more homogeneous texture, and lower strength suggests a more heterogeneous and complex texture [15,28]. This was demonstrated in Figure 3 and highlights a crucial aspect of radiomics, through its capacity to quantify features that are imperceptible through visual inspection yet may be critical in predicting clinical outcome.

Our findings contribute to the growing body of evidence supporting the integration of radiomic factors into existing clinical models for RPS management [19]. While limited research explores the combined use of radiomic and clinical data to predict RPS relapse and survival, the large, multi-cohort RADSARC-S study evaluated the ability of radiomic features to predict histology and grade in patients with primary leiomyosarcoma or liposarcoma RPS undergoing surgical resection. Although the RADSARC-S study successfully predicted grade and histology with high accuracy, it did not assess the model’s ability to predict recurrence or survival [19]. Several other studies investigating non-RPS have reported a similar improved predictive utility of radiomics for relapse, progression, and survival [11,29,30,31,32,33].

Radiomics has the potential to influence treatment algorithms for patients with localized RPS, enabling treatment de-escalation or escalation based on specific features. Currently, treatment decisions typically rely on clinicopathological findings, which are often limited by small biopsy samples that may not capture tumour heterogeneity. In contrast, CT scans, universally employed in RPS workup, allow for the extraction of radiomic features from the entire tumour, potentially enhancing the accuracy of outcome prediction, as demonstrated in our study. Importantly, while the STRASS study did not yield conclusive results, radiomics could identify RPS patients who might benefit from neoadjuvant radiotherapy. Additionally, kurtosis showed utility in predicting time to distant metastasis, potentially identifying RPS patients who could benefit from neo/adjuvant chemotherapy. However, further validation studies and model development for routine clinical practice are warranted.

Our study’s strength lies in combining clinicopathological and radiomics data to enhance the prediction of OS and time to distant metastasis. Additionally, as a real-world study, our patient population may better reflect everyday clinical practice. However, all patients in our cohort were treated before the results of the STRASS trial, meaning they all received neoadjuvant radiotherapy, which may not represent current practices.

Regarding our study limitations, the retrospective nature of this study may have introduced bias, and the modest sample size with limited follow-up and few outcome events only enabled a highly exploratory analysis of radiomic features. No internal or external validation was performed, and radiomic features were assessed individually rather than in combination due to the limited event count. All images were acquired with the same tube potential, without intravenous contrast, and the vast majority of images were acquired with the same scanner and acquisition/reconstruction protocols. This relative homogeneity may limit generalizability to more variation in imaging acquisition protocols such as those with intravenous contrast. Segmentation was performed by experienced operators, but test–retest or interobserver reproducibility was not formally evaluated. No image harmonization or normalization techniques were applied, consistent with prior exploratory single-centre studies. Some hazard ratio estimates were associated with wide confidence intervals, reflecting the limited number of outcome events and small sample size; these should be interpreted as indicating the direction and relative strength of association rather than precise quantitative effects. Given these limitations, no multiplicity correction was performed, and the findings should be considered hypothesis-generating, warranting further validation in larger, prospective multicentre cohorts.

## 5. Conclusions

We identified similar clinicopathological factors associated with time to distant recurrence and OS that was in line with the published literature. Some radiomic features may complement clinical predictors to clinicopathological factors to predict OS and time to distant metastasis in patients with high-risk RPS. These findings are hypothesis-generating and warrant further research for validation and development of tools to assist in treatment decision-making.

## Figures and Tables

**Table 1:** Patient characteristics.

	Risk Group N (%)		Total N(%)
Characteristic	High-Risk (n = 42)	Low-Risk (n = 30)	Total (n = 72)
**Sex**			
Female	15 (36%)	13 (43%)	28 (39%)
Male	27 (64%)	17 (57%)	44 (61%)
**Age**			
Mean (SD)	57 (13)	58 (13)	57 (13)
Median [range]	61 [31–86]	60 [33–82]	60 [31–86]
IQR	48–66	50–66	49–66
**ECOG**			
0	23 (55%)	19 (63%)	42 (58%)
1	19 (45%)	10 (33%)	29 (40%)
2	0	1 (3%)	1 (1%)
**Max dimension on imaging, mm**			
Mean (SD)	155 (81)	130 (62)	144 (74)
Median [range]	136 [40–360]	130 [23–270]	132 [23–360]
IQR	90–198	77–178	88–190
**Grade**			
1	1 (3%)	22 (79%)	23 (35%)
2	19 (51%)	5 (18%)	24 (37%)
3	17 (46%)	1 (4%)	18 (28%)
Missing	5	2	7
**Histologic subtype**			
Well-differentiated liposarcoma	0	15 (50%)	15 (21%)
Leiomyosarcoma	7 (17%)	3 (10%)	10 (14%)
Solitary fibrous tumour	0	8 (27%)	8 (11%)
Undifferentiated pleomorphic sarcoma	5 (12%)	0	5 (7%)
Well-diff/de-differentiated liposarcoma	30 (71%)	0	30 (42%)
Other	0	4 (13%)	4 (6%)
**Multifocal**			
No	39 (93%)	29 (97%)	68 (94%)
Yes	3 (7%)	1 (3%)	4 (6%)

**Table 2:** Cox-proportional hazard model for OS and clinical variables.

Overall Survival							
				Univariable		Multivariable	
Variable	Level	N	Events	HR (95% CI)	*p*-Value	HR (95% CI)	*p*-Value
Age	Per 5 years increase	72	18	1.2 (1.0, 1.4)	0.125	1.3 (1.0, 1.8)	0.040
Max dimension on imaging	Per 10 cm increase	72	18	1.5 (0.8, 2.6)	0.176	4.0 (1.2, 13.0)	0.015
Grade	1	23	1	ref	0.004	ref	0.002
	2	24	6	5.3 (0.6, 44.4)		22.0 (1.5, 323.9)	
	3	18	8	15.1 (1.8, 124.8)		180.3 (6.8, 4802.7)	
Subtype risk group	High-risk	42	15	ref	0.023	ref	0.114
	Low-risk	30	3	0.3 (0.1, 0.9)		5.1 (0.8, 33.4)	

**Table 3:** Overall survival by radiomics features adjusted by age and tumour size in the high-risk subset of patients.

			Adjusted by Age and Max Dimension on Imaging		
Category	Variable	Level	HR (95% CI)	*p*-Value	C-Statistic
First Order	10th percentile	Per 10 increase	1.1 (0.9, 1.4)	0.309	0.65
	90th percentile	Per 10 increase	1.2 (0.7, 2.1)	0.430	0.64
	**Kurtosis**	**Per 100 increase**	**0.1 (0.0, 9.3)**	**0.013**	**0.69**
	Minimum	Per 100 increase	1.1 (0.8, 1.4)	0.606	0.61
	Skewness	Per 1 increase	1.0 (0.8, 1.1)	0.429	0.62
GLCM	**Cluster shade**	**Per 1000 increase**	**0.4 (0.0, 8.2)**	**0.062**	**0.69**
	**IDN**	**Per 0.01 increase**	**0.6 (0.3, 1.0)**	**0.062**	**0.74**
	**IMC1**	**Per 0.01 increase**	**1.1 (1.0, 1.2)**	**0.128**	**0.72**
	Inverse variance	Per 0.01 increase	1.0 (0.9, 1.2)	0.739	0.58
	Max probability	Per 0.01 increase	1.0 (0.9, 1.1)	0.511	0.62
GLDM	**Dependence variance**	**Per 1 increase**	**0.9 (0.8, 1.1)**	**0.254**	**0.68**
	**Gray level variance**	**Per 10 increase**	**0.6 (0.3, 1.4)**	**0.082**	**0.68**
GLRLM	Long run low gray level emphasis	Per 0.01 increase	0.8 (0.3, 1.9)	0.600	0.63
GLSZM	**Gray level non-uniformity**	**Per 1000 increase**	**1.2 (1.0, 1.4)**	**0.098**	**0.71**
	**Gray level variance**	**Per 100 increase**	**0.5 (0.1, 2.9)**	**0.077**	**0.67**
	Large area emphasis	Per 1,000,000 increase	1.0 (0.9, 1.0)	0.671	0.59
	Large area low gray level emphasis	Per 10,000 increase	1.2 (0.9, 1.7)	0.179	0.65
	Size zone non-uniformity normalized	Per 0.01 increase	1.0 (0.8, 1.1)	0.572	0.62
	**Zone entropy**	**Per 1 increase**	**0.5 (0.1, 1.9)**	**0.261**	**0.68**
	Zone percentage	Per 0.01 increase	1.0 (0.8, 1.3)	0.964	0.58
NGTDM	**Busyness**	**Per 100 increase**	**2.4 (1.2, 4.8)**	**0.036**	**0.73**
	Contrast	Per 0.01 increase	0.7 (0.1, 5.3)	0.723	0.61
	**Strength**	**Per 1 increase**	**0.1 (0.0, 47.8)**	**0.036**	**0.72**
Shape	Elongation	Per 0.1 increase	1.0 (0.6, 1.8)	0.891	0.59
	**Flatness**	**Per 0.1 increase**	**1.3 (0.8, 2.1)**	**0.364**	**0.67**
	Major axis length	Per 100 increase	1.5 (0.4, 4.8)	0.544	0.62
	Sphericity	Per 0.1 increase	0.8 (0.4, 1.7)	0.594	0.57

Bold rows indicate radiomic features with an increase in c-statistic > 0.1 compared with the clinical model including age and tumor size alone

## Data Availability

The data presented in this study are not publicly available due to their containing information that could compromise the privacy of research participants but are available from the corresponding author.

## Supplementary Materials

The following supporting information can be downloaded at: https://www.mdpi.com/article/10.3390/jimaging11120450/s1, Supplementary Table S1: Radiomic features evaluated in the study; Supplementary Table S2: Distribution of histologic subtype and tumour grade within the study cohort; Supplementary Table S3: Univariable and multivariable Cox proportional hazards analysis for relapse-free survival; Supplementary Table S4: Summary of radiomic features associated with time to distant metastasis (TDM).

## Abbreviation

RPS	Retroperitoneal sarcoma
STS	Soft-tissue sarcoma
UPS	Undifferentiated pleomorphic sarcoma
WD-DDLPS	Well-differentiated/De-differentiated liposarcoma
LMS	Leiomyosarcoma
OS	Overall survival
RFS	Relapse-free survival
TLR	Time to local recurrence
TDM	Time to distant metastasis
HR	Hazard ratio
CI	Confidence interval
CT	Computed tomography
RT	Radiotherapy
GTV	Gross tumour volume
ROI	Region of interest
GLCM	Gray level co-occurrence matrix
GLDM	Gray Level Dependence Matrix
GLRLM	Gray Level Run Length Matrix
GLSZM	Gray Level Size Zone Matrix
NGTDM	Neighbourhood Gray-Tone Difference Matrix
IDN	Inverse difference normalized
IMC1	Informational measure of correlation 1
IQR	Interquartile range
ECOG	Eastern Cooperative Oncology Group (Performance Status)
C-statistic	Concordance statistic
QA	Quality assurance
STRASS	EORTC-62092: Preoperative Radiotherapy Plus Surgery Versus Surgery Alone for Primary Retroperitoneal Sarcoma
ANZSA	Australian and New Zealand Sarcoma Association
