# Supplementary material for: Applying Radiomics to Predict Outcomes in Patients with High-Grade Retroperitoneal Sarcoma Treated with Preoperative Radiotherapy
Source: J Imaging. 2025 Dec 15;11(12):450. doi: 10.3390/jimaging11120450 (PMC12733741; doi:10.3390/jimaging11120450)
Supplement: Supplementary file 1 [file jimaging-11-00450-s001.zip › jimaging-3946210-supplementary.pdf]

Supplementary Table 1. Radiomic features that were evaluated

Radiomic Feature
original_shape_LeastAxisLength
original_shape_Maximum3DDiameter
original_shape_Sphericity
original_shape_Maximum2DDiameterRow
original_shape_SurfaceVolumeRatio
original_shape_Elongation
original_shape_MinorAxisLength
original_shape_VoxelVolume
original_shape_MajorAxisLength
original_shape_Maximum2DDiameterColumn
original_shape_Flatness
original_shape_MeshVolume
original_shape_Maximum2DDiameterSlice
original_shape_SurfaceArea
original_gldm_DependenceNonUniformityNormalized
original_gldm_DependenceVariance
original_gldm_HighGrayLevelEmphasis
original_gldm_LargeDependenceLowGrayLevelEmphasis
original_gldm_GrayLevelNonUniformity
original_gldm_SmallDependenceHighGrayLevelEmphasis
original_gldm_LowGrayLevelEmphasis
original_gldm_LargeDependenceHighGrayLevelEmphasis
original_gldm_LargeDependenceEmphasis
original_gldm_SmallDependenceLowGrayLevelEmphasis
original_gldm_GrayLevelVariance
original_gldm_DependenceNonUniformity
original_gldm_SmallDependenceEmphasis
original_gldm_DependenceEntropy
original_glcm_Idn
original_glcm_Correlation
original_glcm_SumSquares
original_glcm_ClusterProminence
original_glcm_InverseVariance
original_glcm_ClusterTendency
original_glcm_Imc1
original_glcm_DifferenceEntropy
original_glcm_MaximumProbability
original_glcm_JointAverage
original_glcm_Autocorrelation

original_glcm_JointEntropy
original_glcm_Idm
original_glcm_DifferenceVariance
original_glcm_JointEnergy
original_glcm_SumEntropy
original_glcm_Idmn
original_glcm_Contrast
original_glcm_Imc2
original_glcm_DifferenceAverage
original_glcm_ClusterShade
original_glcm_Id
original_ngtdm_Contrast
original_ngtdm_Busyness
original_ngtdm_Complexity
original_ngtdm_Strength
original_ngtdm_Coarseness
original_glrlm_ShortRunHighGrayLevelEmphasis
original_glrlm_RunEntropy
original_glrlm_GrayLevelVariance
original_glrlm_ShortRunEmphasis
original_glrlm_LongRunEmphasis
original_glrlm_HighGrayLevelRunEmphasis
original_glrlm_GrayLevelNonUniformity
original_glrlm_ShortRunLowGrayLevelEmphasis
original_glrlm_GrayLevelNonUniformityNormalized
original_glrlm_RunVariance
original_glrlm_RunLengthNonUniformity
original_glrlm_RunLengthNonUniformityNormalized
original_glrlm_RunPercentage
original_glrlm_LongRunLowGrayLevelEmphasis
original_glrlm_LongRunHighGrayLevelEmphasis
original_glrlm_LowGrayLevelRunEmphasis
original_firstorder_Energy
original_firstorder_Maximum
original_firstorder_Median
original_firstorder_Skewness
original_firstorder_Mean
original_firstorder_MeanAbsoluteDeviation
original_firstorder_10Percentile
original_firstorder_90Percentile
original_firstorder_Kurtosis
original_firstorder_RootMeanSquared
original_firstorder_RobustMeanAbsoluteDeviation

original_firstorder_Range
original_firstorder_Variance
original_firstorder_TotalEnergy
original_firstorder_Uniformity
original_firstorder_Minimum
original_firstorder_InterquartileRange
original_firstorder_Entropy
original_glszm_LargeAreaEmphasis
original_glszm_GrayLevelVariance
original_glszm_ZoneEntropy
original_glszm_HighGrayLevelZoneEmphasis
original_glszm_GrayLevelNonUniformity
original_glszm_LargeAreaLowGrayLevelEmphasis
original_glszm_SizeZoneNonUniformity
original_glszm_ZonePercentage
original_glszm_SmallAreaEmphasis
original_glszm_GrayLevelNonUniformityNormalized
original_glszm_ZoneVariance
original_glszm_SmallAreaLowGrayLevelEmphasis
original_glszm_SizeZoneNonUniformityNormalized
original_glszm_LargeAreaHighGrayLevelEmphasis
original_glszm_LowGrayLevelZoneEmphasis
original_glszm_SmallAreaHighGrayLevelEmphasis

Supplementary Table 2. The distribution of grade and subtype within the cohort

**Histologic subtype with grade**	**High risk (n = 42)**	**Low risk (n = 30)**	**Total (n = 72)**
Well-differentiated liposarcoma (Grade 1)	0	15 (50%)	15 (21%)
Leiomyosarcoma (missing grade)	0	1 (3%)	1 (1%)
Leiomyosarcoma (Grade 1)	0	2 (7%)	2 (3%)
Leiomyosarcoma (Grade 2)	4 (10%)	0	4 (6%)
Leiomyosarcoma (Grade 3)	3 (7%)	0	3 (4%)
Other (Grade 1)	0	2 (7%)	2 (3%)
Other (Grade 2)	0	2 (7%)	2 (3%)
Solitary fibrous tumour (missing grade)	0	1 (3%)	1 (1%)
Solitary fibrous tumour (Grade 1)	0	3 (10%)	3 (4%)

Histologic subtype with grade	High risk (n = 42)	Low risk (n = 30)	Total (n = 72)
Solitary fibrous tumour (Grade 2)	0	3 (10%)	3 (4%)
Solitary fibrous tumour (Grade 3)	0	1 (3%)	1 (1%)
Undifferentiated pleomorphic sarcoma (Grade 2)	1 (2%)	0	1 (1%)
Undifferentiated pleomorphic sarcoma (Grade 3)	4 (10%)	0	4 (6%)
Well-diff/de-differentiated liposarcoma (missing grade)	5 (12%)	0	5 (7%)
Well-diff/de-differentiated liposarcoma (Grade 1)	1 (2%)	0	1 (1%)
Well-diff/de-differentiated liposarcoma (Grade 2)	14 (33%)	0	14 (19%)
Well-diff/de-differentiated liposarcoma (Grade 3)	10 (24%)	0	10 (14%)

Supplementary Table 3. Summary of the Cox-proportional hazard analysis for relapse free survival

				Univariable		Multivariable	
**Variable**	**Level**	**N**	**Events**	**HR (95% CI)**	**p-value**	**HR (95% CI)**	**p-value**
Age	Per 5 years increase	72	28	1.2 (1.0, 1.4)	0.044	1.3 (1.0, 1.6)	0.011
Max dimension on imaging	Per 10 cm increase	72	28	0.9 (0.6, 1.6)	0.837	1.1 (0.5, 2.5)	0.811
Grade	1	23	3	ref	0.002	ref	0.073
	2	24	9	3.3 (0.9, 12.1)		4.3 (0.7, 25.4)	
	3	18	11	7.2 (2.0, 26.1)		10.6 (1.4, 82.6)	
Subtype risk group	High risk	42	22	ref	0.011	ref	0.727
	Low risk	30	6	0.3 (0.1, 0.8)		1.3 (0.3, 6.6)	

Supplementary Table 4. Summary of TDM radiomic features

Category	Variable	Level	Adjusted by Age and Max dimension on imaging		
			HR (95% CI)	p-value	C-statistic
First Order	10 percentile	Per 10 increase	1.1 (0.9, 1.3)	0.430	0.64
	**90 percentile**	**Per 10 increase**	**1.5 (0.8, 2.7)**	**0.198**	**0.72**
	**Kurtosis**	**Per 100 increase**	**0.1 (0.0, 10.3)**	**0.023**	**0.72**
	Minimum	Per 100 increase	1.1 (0.8, 1.4)	0.657	0.62
	Skewness	Per 1 increase	1.0 (0.8, 1.1)	0.616	0.63
GLCM	Cluster shade	Per 1000 increase	1.0 (0.9, 1.0)	0.302	0.66
	IDN	Per 0.01 increase	0.7 (0.4, 1.3)	0.261	0.69
	IMC1	Per 0.01 increase	1.1 (1.0, 1.2)	0.232	0.69
	Inverse variance	Per 0.01 increase	1.0 (0.8, 1.2)	0.910	0.60
	Max probability	Per 0.01 increase	0.9 (0.8, 1.1)	0.273	0.67
GLDM	**Dependence variance**	**Per 1 increase**	**0.9 (0.8, 1.0)**	**0.217**	**0.70**
	Gray level variance	Per 10 increase	0.9 (0.6, 1.2)	0.359	0.66
GLRLM	Long run low gray level emphasis	Per 0.01 increase	0.7 (0.3, 1.6)	0.385	0.62
GLSZM	**Gray level non-uniformity**	**Per 1000 increase**	**1.2 (1.0, 1.4)**	**0.141**	**0.74**
	Gray level variance	Per 100 increase	0.8 (0.5, 1.3)	0.288	0.66
	Large area emphasis	Per 1000000 increase	1.0 (0.9, 1.0)	0.314	0.62
	Large area low gray level emphasis	Per 10000 increase	1.1 (0.8, 1.5)	0.459	0.65
	Size zone non-uniformity normalised	Per 0.01 increase	1.0 (0.9, 1.2)	0.924	0.60
	Zone entropy	Per 1 increase	0.9 (0.3, 3.0)	0.814	0.60
	Zone percentage	Per 0.01 increase	1.0 (0.8, 1.3)	0.689	0.63
NGTDM	**Busyness**	**Per 100 increase**	**1.9 (1.0, 3.5)**	**0.095**	**0.71**
	Contrast	Per 0.01 increase	0.7 (0.1, 4.9)	0.703	0.61
	Strength	Per 1 increase	1.0 (0.9, 1.1)	0.252	0.66
Shape	Elongation	Per 0.1 increase	0.8 (0.5, 1.3)	0.377	0.64
	Flatness	Per 0.1 increase	0.9 (0.5, 1.5)	0.642	0.60
	Major axis length	Per 100 increase	2.3 (0.6, 9.6)	0.234	0.65
	Sphericity	Per 0.1 increase	0.6 (0.3, 1.4)	0.236	0.64
